# The Leper Hospitals of the West Indies

**Published:** 1898-07-02

**Authors:** Louis Vintras

**Affiliations:** Paris, Physician to the French Hospital


### THE LEPER HOSPITALS OF THE WEST INDIES.

By Louis Vintras, M.D. Durh., B.Sc. Paris, Physician to the French Hospital.

I.

The numerous affinities which certainly exist between leprosy and tuberculosis made me wish, during my stay in the West Indies, to see as much as possible of the former disease; this led to my visiting, whenever practicable, the various leper establishments, and making a thorough investigation into the conditions under which the unfortunate people suffering from this loathsome malady live. I must say at the start that in most cases the care taken of these poor people is very creditable to the Governments concerned, and the way in which several of these establishments are administered would do credit to many of our charitable institutions at home. This is all the more praiseworthy to those concerned that private charity is here quite out of the question, and that everything is done by public money and through official channels. State charity can never afford to be over-generous, and has mostly to content itself with the supply of necessities, but in the case of such patients as these, condemned to carry their burden of bodily troubles through lengthy years of cheerless life, what a world of meaning lies in the smallest attempt at comfort.

Unfortunately, at the present moment the West Indies are in the throes of the worst financial crisis yet experienced by these heretofore lucky colonies, and instead of new monetary outlays being contemplated, it is the existent expenditure that has everywhere to be cut down. Hence it is that in several places a state of affairs is allowed to exist in certain of the less conspicuous parts of the Government services which would not compare favourably with the administrative organisations of a second-rate Central American republic.

Lepers are a class of individuals who do not appeal very strongly to the public imagination, and so long as they are kept well out of sight no one takes very much heed as to their mode of life. It is easy to understand, then, that these unfortunate people would be the last to benefit from any budgetary increase, and the first to suffer from administrative economy.

Thus it is that of late years very little has been done for the West Indian leper, and where any improvement has taken place in his hospital life, it is due entirely to the zeal and good management of those in charge of the several establishments, but not to any interest shown by the various Governments. These seem to remain content with the first outlay, and in several cases there is little to find fault with; but in others, when the primary organisation was shockingly deficient, the same cannot be said.

The chief aim of the authorities appears to be the strict isolation of lepers, and all their efforts are being now expended in that direction. This is well in accordance with the very latest ideas, and, admitting the unmistakable affinities between leprosy and tuberculosis, is only the direct outcome of ideas held at home in connection with the last-named disease. But however sound these ideas may seem in the abstract, it must be admitted that they are somewhat incongruous when put into practice. The most contradictory statements are made as to the contagion of this disease even by professional men; and, while in one place you are told that it is dangerous to purchase baskets made by lepers, in others you hear that healthy females have intercourse with lepers without any untoward results. At present, however, we have only to do with the leper establishments, and this is not the place to discuss a question involved in so much uncertainty; but, in the face of such conflicting evidence, it may be allowed us to deplore that the tendency of the authorities should be to think less of the welfare of the patients than of their segregation. "Let us do everything that we can to make them happy and to ameliorate their sad condition, but for the sake of the well members of the community let us do all we can to get the lepers to go into the lazaretto," says Dr. C. Hutson, the poor-law inspector for Barbados, in his annual report for 1897, and with this the Governments generally agree, but find the second proposition less expensive to carry out than the first.

Since I have mentioned Dr. C. Hutson, by whose kindness and courtesy I was enabled to visit the Barbados Lazaretto, I will begin with this institution. Situated on the slope of a hill about half an hour's drive from the centre of Bridgetown, the lazaretto is composed of a series of low buildings, one storey high, and rather cramped together on a plot of ground altogether inadequate for a community of people averaging one hundred and eleven, without counting the *personnel*. The houses when first built consisted only of a ground floor, but another storey has been added in parts; they are built on the principle of most West Indian houses, a covered verandah closed in with Spanish windows running along the front and back. The front verandah is the inmates' common-room, and here they take their meals, while the wards open on the verandah. Some of the wards run the whole width of the building, while others are much smaller, containing only six beds; all are too low in the ceiling and have a stuffy, oppressive appearance, while in the smaller rooms the ventilation is inadequate. The wards on the first floor are more airy and much brighter, and possess a simple and excellent system of ventilation. The walls and ceilings are whitewashed, the floors are of plain wood, and everything was clean and in good order in the wards, which speaks all the more for those in charge of them that the visit Dr. Hutson and I paid the institution was quite unexpected. I cannot say the same of the kitchen, which is located in an outhouse, and where everything was dirty, the cooks included. The food which was then being prepared for the midday meal was wholesome enough, but was not prepared with care, nor had it a very appetising appearance. The water-closets, which are situated at the bottom of the garden, are on the earth system, and for such a climate I know of no better system. A row of seats runs on each side of this shed, the seats are quite open, and, though it was already midday, there was not the slightest smell, and this was equally true of the surroundings. The pans are removed every morning, emptied in the garden, and replenished with new earth. The garden, however, is much too small for the purpose, and this continued deposit of contaminated earth, though so far causing no nuisance, cannot be considered healthy.

The patients are clean, and appear cheerful; the women are more easy to deal with in respect to cleanliness than the men, who are both dirty in habits and in their surroundings. This is one of the great drawbacks of a demoralising disease, and it is only too evident to those who have been among them that they have come to consider themselves as outcasts, and gradually lose all self-respect. I think that this is one of the greatest difficulties to be found in any endeavour to stamp out the disease, and unfortunately there has been little in their surroundings so far to counteract this tendency. Enough opportunities are not afforded, to the men especially, to indulge in useful or interesting occupations. Some of them are exceedingly clever, and, when means are given them to exercise their abilities, they do so willingly, and there are in this institution many curious examples of what they can do with only the rawest of materials. The women are occupied with the washing, ironing, and some of the household duties, and are, on the whole, much brighter and much less dissatisfied than the men. The sexes are, of course, strictly separated.

From the half-yearly report for 1897 (January-June) I find that during that period 115 lepers were treated, the actual number resident having increased from 107 to 112. An average of 30 inmates have been employed at work. The expenditure for the half-year was £1,086 4s. 8d., the daily average cost per head for feeding alone being 6d.

On the whole the lazaretto is well managed, though in some matters economy seems to border on parsimony, and the buildings themselves, though old, could still be made very comfortable if not quite so crowded, and if, by the addition of another plot of land, more accommodation could be given the patients for exercise. The place wants more air, both within and without, and at present savours more of the prison than the hospital.

